# Effect of Different Solvents on Morphology and Gas-Sensitive Properties of Grinding-Assisted Liquid-Phase-Exfoliated MoS_2_ Nanosheets

**DOI:** 10.3390/nano12244485

**Published:** 2022-12-18

**Authors:** Hao Wang, Xiaojie Xu, Talgar Shaymurat

**Affiliations:** 1 Key Laboratory of New Energy and Materials Research, Xinjiang Institute of Engineering, Urumqi 830023, China; 2 Xinjiang Condensed Matter Phase Transition and Microstructure Laboratory, College of Physics Science and Technology, Yili Normal University, Yining 835000, China

**Keywords:** two-dimensional materials, MoS_2_ nanosheets, liquid-phase exfoliation, grinding solvent, gas-sensitive properties

## Abstract

Grinding-assisted liquid-phase exfoliation is a widely used method for the preparation of two-dimensional nanomaterials. In this study, N-methylpyrrolidone and acetonitrile, two common grinding solvents, were used during the liquid-phase exfoliation for the preparation of MoS_2_ nanosheets. The morphology and structure of MoS_2_ nanosheets were analyzed via scanning electron microscopy, X-ray diffraction, and Raman spectroscopy. The effects of grinding solvents on the gas-sensing performance of the MoS_2_ nanosheets were investigated for the first time. The results show that the sensitivities of MoS_2_ nanosheet exfoliation with N-methylpyrrolidone were 2.4-, 1.4-, 1.9-, and 2.7-fold higher than exfoliation with acetonitrile in the presence of formaldehyde, acetone, and ethanol and 98% relative humidity, respectively. MoS_2_ nanosheet exfoliation with N-methylpyrrolidone also has fast response and recovery characteristics to 50–1000 ppm of CH_2_O. Accordingly, although N-methylpyrrolidone cannot be removed completely from the surface of MoS_2_, it has good gas sensitivity compared with other samples. Therefore, N-methylpyrrolidone is preferred for the preparation of gas-sensitive MoS_2_ nanosheets in grinding-assisted liquid-phase exfoliation. The results provide an experimental basis for the preparation of two-dimensional materials and their application in gas sensors.

## 1. Introduction

Given the special structure and potential applications, two-dimensional (2D) materials such as graphene, boron nitride, and molybdenum disulfide (MoS_2_) draw plenty of concerns. Among them, MoS_2_ as the frontrunner in transition metal dichalcogenides (TMDCs) materials has gained the most attention [1,2,3,4] and is used in a wide variety of applications [5,6,7,8,9,10,11] due to its unique properties [12,13,14]. MoS_2_ is at the forefront in the race of an ideal gas-sensing material because of its large surface-to-volume ratio, enormous number of active sites, and favorable adsorption sites [15,16]. MoS_2_ manifests two possible crystal phases, including trigonal and hexagonal structures, with metallic and semiconducting properties, respectively [17]. The presence of weak Van der Waals force facilitates the isolation of layers from bulk MoS_2_. The indirect bandgap of 1.2 eV in bulk MoS_2_ is converted to a direct bandgap of 1.8 eV for monolayer MoS_2_ [3,14,18]. The absence of dangling bonds provides stability to pristine MoS_2_ flakes in liquid and gaseous media in the presence of oxygen, thereby facilitating its gas-sensing application [19,20]. Therefore, a reliable and low-cost technique is needed to produce 2D-MoS_2_ for gas-sensing applications. Currently, several methods including vapor deposition [21], mechanical exfoliation [22], lithium-ion intercalation [23], liquid-phase exfoliation [24,25], and RF sputtering [26] have been utilized to fabricate 2D-MoS_2_ nanomaterials. Although high-quality MoS_2_ nanosheets were prepared by mechanical methods for fundamental research, it is difficult to meet the need for mass production. Meanwhile, hydrothermal and solvothermal methods yield few-layer MoS_2_ nanosheets on a large scale. However, they often require high temperature and high pressure. Lithium intercalation into the layered structure of 2D-MoS_2_ is limited by long lithiation time, high temperature, and sensitivity to environmental conditions. Instead, grinding-assisted liquid-phase exfoliation is not air-sensitive, does not entail chemical reaction, and generally has acceptable yield [3,27,28]. Coleman et al. [29] evaluated multiple solvents for ultrasonic exfoliation of materials. They found that the most effective solvent was N-methylpyrrolidone (NMP), followed by acetonitrile (ACN). Yao et al. [25] reported that relatively high yields up to 26.7 mg/mL were obtained by incorporating NMP as a grinding solvent into the exfoliation procedure because the surface energy of NMP is similar to that of MoS_2_. As a result, NMP is the preferred solvent in liquid-phase exfoliation to obtain single or multilayered 2D nano-materials [25,27,28,29,30,31].

The studies suggest that grinding solvent plays an important role in grinding-assisted exfoliation because its physical properties, such as boiling point [32], surface tension and energy [29,33], as well as solubility parameters [24], can affect the final exfoliation materials. Emily et al. reported that the lateral size and thickness of the exfoliated flakes of MoS_2_ nanosheets are highly dependent on the solvent. NMP yielded flakes of the highest quality based on lateral size and flake thickness. The NMP remained on the surface of the MoS_2_ nanosheets when ACN was completely removed [34]. Good yields were obtained when using NMP as a grinding solvent. However, whether the NMP residue affects the performance of electronic devices is unknown. It may adversely affect the application of MoS_2_ nanosheets in gas sensing. To our knowledge, there is no report on the research of the effects of a grinding solvent on the gas-sensing properties of MoS_2_. 

Therefore, we evaluated the effects of residual NMP on the morphology and gas-sensing properties of liquid-phase-exfoliated MoS_2_ nanosheets. We selected ACN, which has a lower boiling point and easier solvent removal compared with NMP. The morphology and structure of MoS_2_ nanosheets were analyzed by scanning electron microscopy (SEM), X-ray diffraction (XRD), and Raman spectroscopy. The effects of grinding solvents on the gas-sensing performance of MoS_2_ nanosheets were investigated for the first time. 

## 2. Materials and Methods

### 2.1. Preparation of Materials

MoS_2_, with a purity of 99% and particle size less than 2 μm, was purchased from Sigma-Aldrich. ACN, NMP, and absolute ethanol (C_2_H_6_O) were purchased from Tianjin Zhiyuan Chemical Reagent Co. Ltd. as analytically pure reagents. The preparation of MoS_2_ nanosheets via grinding-assisted liquid-phase exfoliation is described as follows:

MoS_2_ powder (100 mg) was manually ground in a mortar for 2 h, and 0.5 mL of the chosen solvent was added during the grinding. The sample was then dried in a vacuum oven at 60 °C for 12 h. The dried sample was dispersed in 40 mL of 45 vol% absolute ethanol and sonicated for 1 h at 120 W with stirring. The dispersion was centrifuged for another 20 min (1500 r / min) to obtain the MoS_2_ nanosheets, and the supernatant was dried in air for further use. For convenience, the MoS_2_ nanosheets obtained by grinding with ACN were designated as S1, and those ground with NMP were called S2.

### 2.2. Characterizations

The morphology of MoS_2_ nanosheets was observed with a field emission scanning electron microscope (SEM, JSM-7610F Plus). The crystal structure of MoS_2_ nanosheets was characterized by X-ray diffraction (XRD, Bruker D8 Advance, with Cu-Kα radiation). Raman spectroscopy (Renishaw inVia, Gloucester, Britain) was used to characterize the defects and functional groups of samples. The I-t and I-V curves of the sensing chip were measured by Keithley 2636B at room temperature.

### 2.3. Device Fabrication and Testing

The MoS_2_ nanosheets were dispersed in absolute ethanol at 10 mg/mL. Dispersions (2 μL) were uniformly coated to fabricate a MoS_2-_based sensing chip with Ag-Pd fork-finger electrodes. The minimum width and spacing of electrodes was 0.2 mm. The interdigital electrode was dried at 25 °C and aged for 24 h at a voltage of 4 V to obtain a sensing chip with good stability. The target vapor was produced by thermal evaporation, according to our previous work [35], and a calculated amount of target liquid was dropped onto a hot plate in a 1 L container to generate target vapor in the container. Next, 98% relative humidity was obtained by saturating salt solution (potassium sulphate-K₂SO₄). Then, by transferring the sensing chip from the air to the target gas at room temperature, the Keithley 2636B recorded the change of the current signal of the sensing chip (Figure S1). The response was defined using the formula $\left(\frac{I_{G}-I_{R}}{I_{R}}\right)\times 100\%$, where *I_R_* and *I_G_* are the currents of the sensor in the reference gas and target gas, respectively. The response time and recovery time were defined as the response values of 90% and 10% of the current of the sensor in contact with the target gas, respectively.

## 3. Results and Discussion

**Figure 1:**
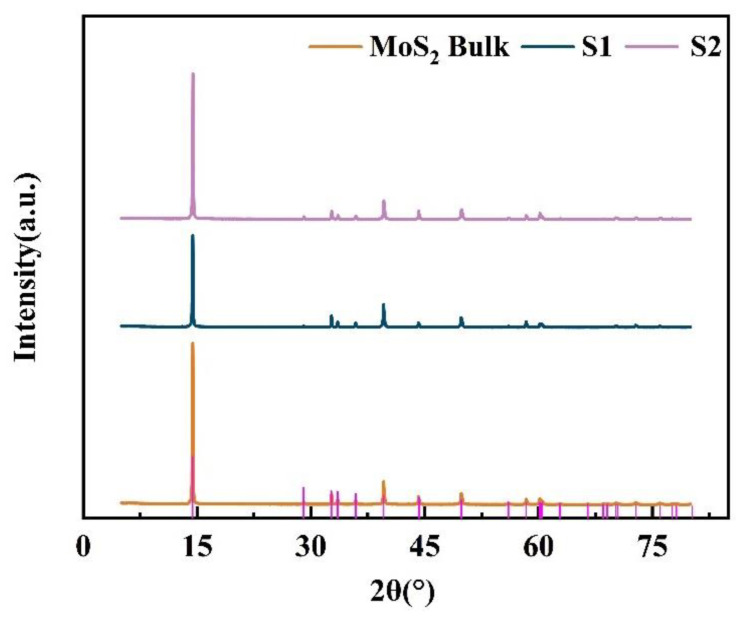
XRD patterns of the bulk MoS_2_: S1 and S2.

The XRD patterns of the two types of MoS_2_ prepared by different grinding solvents are shown in Figure 1. Compared with JCPDS Card No. 73-1508, the lattice constants were: a = 3.15938 Å, b = 3.15938 Å, and c = 12.28962 Å. The diffraction peaks 14.39°, 29.02°, 32.69°, 33.51°, 35.88°, 39.56°, 44.27°, 49.81°, and 56.01° in the figures correspond to (002), (004), (100), (101), (102), (103), (104), (105), and (106) crystal planes of MoS_2_, indicating that the materials were well-crystallized MoS_2_. The peak intensity of MoS_2_ nanosheets weakened, and the FWHM broadening (Figure S2) of the peaks appeared after liquid-phase exfoliation, indicating that the MoS_2_ nanosheets were able to be exfoliated, and thus, the size of MoS_2_ decreases [36,37,38,39].

**Figure 2:**
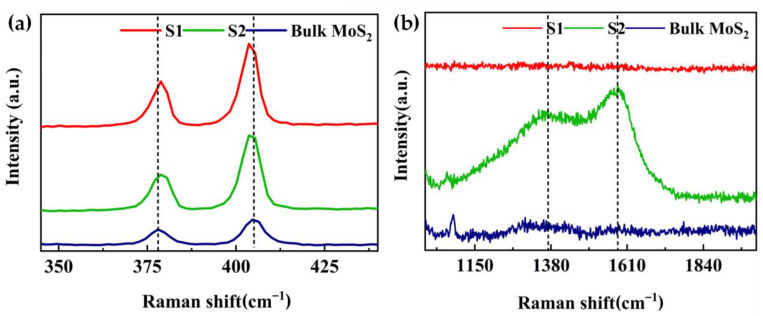
Raman spectra of the bulk MoS_2_, S1 and S2. (**a**) Enlargement of the MoS_2_ peaks $E_{2g}^{1}$ and $A_{1g}$ and (**b**) Enlargement of the MoS_2_ peaks from 1150 cm^−1^ to 1840 cm^−1^.

Raman spectroscopy is effective in distinguishing bulk from exfoliated 2D materials. Figure 2 shows the Raman spectra of bulk MoS_2_: S1 and S2. The two Raman peaks correspond to the high-energy $A_{1g}$ mode and lower-energy $E_{2g}^{1}$ mode. As shown in Figure 2a, all the samples displayed the $E_{2g}^{1}$ and $A_{1g}$ peaks of MoS_2_. Comparing with peaks of bulk MoS_2_, a red shift of $E_{2g}^{1}$ peak and a blue shift of the $A_{1g}$ peak were observed for both S1 and S2, respectively. These shifts are associated with nanosheets obtained with NMP and ACN [40,41]. Figure 2b presents two very broad and intense Raman peaks (1360 and 1580- cm^−1^) of S2, which may be assigned to NMP [31,36] that was not completely removed from the surface of MoS_2_ nanosheets although it was heated and reduced at 60 °C for several hours. In contrast, S1 showed no broad peaks, indicating that ACN was almost removed.

**Figure 3:**
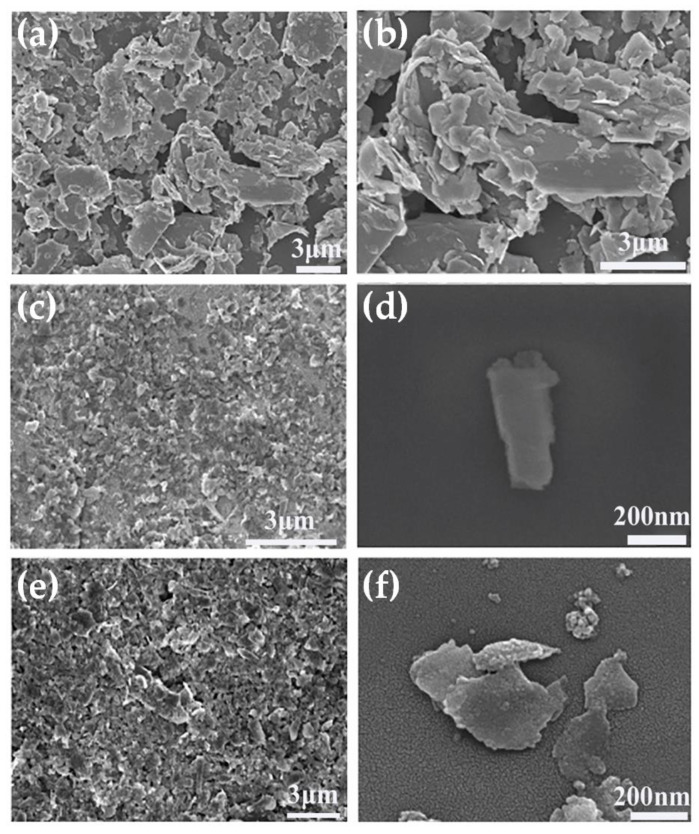
(**a**,**b**) SEM images of bulk MoS_2_. (**c**,**d**) MoS_2_ nanosheets obtained by grinding with ACN (S1). (**e**,**f**) MoS_2_ nanosheets obtained by grinding with NMP (S2).

We next investigated the effect of grinding solvents on the morphology of MoS_2_ nanosheets. The SEM image shown in Figure 3a,b reveals the morphology of the starting MoS_2_ powder as a thick layer with dimensions ranging from about 1 to 6.4 μm. The SEM images presented in Figure 3c,d clearly indicate that the lateral sizes and thicknesses of layered MoS_2_ were reduced by combined grinding and sonication. The MoS_2_ nanosheets were obtained by grinding with ACN (S1), as shown in Figure 3c,d, and the nanosheets were uniform in size and well-dispersed, with the majority measuring between 0.1 and 0.5 μm. As shown in Figure 3e,f, exfoliation with NMP (S2) also produced nanosheets with good dispersion with lateral dimensions of 0.4–1.6 μm. The MoS_2_ nanosheets obtained by grinding with ACN were smaller than NMP-ground MoS_2_ nanosheets, which is consistent with the results reported in the literature [34] and the results of XRD patterns (Figure 1 and Figure S2).

**Figure 4:**
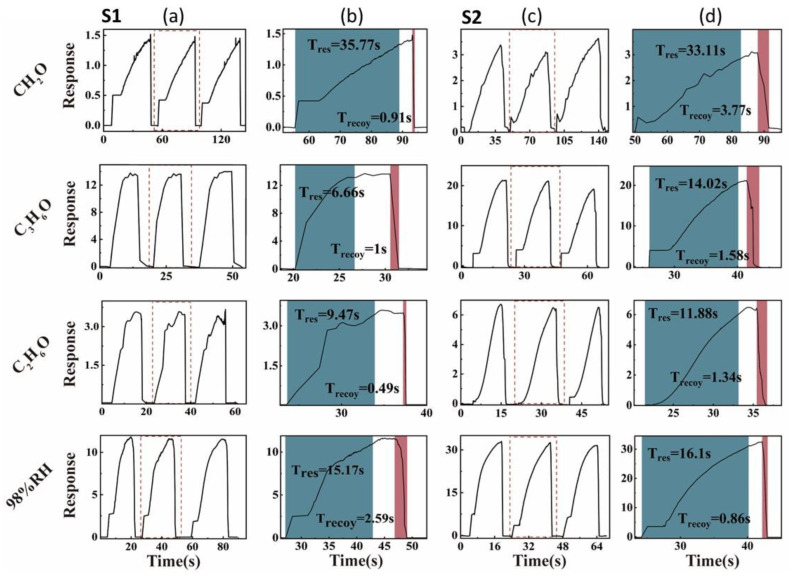
Sensing curves in the presence of different target gases of S1 and S2. (**a**,**c**) Gas-sensitive properties of S1 and S2 at 98% RH and 1000 ppm of CH_2_O, C_3_H_6_O, and C_2_H_6_O, respectively. (**b**,**d**) Response time of S1 and S2 at 98% RH and 1000 ppm of CH_2_O, C_3_H_6_O, and C_2_H_6_O, respectively.

The gas-sensitive properties of MoS_2_ nanosheets loaded on ceramic substrates were tested at room temperature. The results shown in Figure 4a,c indicate gas-sensitive properties and response time (Figure 4b,d) of S1 and S2 at 98% relative humidity (RH) and 1000 ppm of formaldehyde (CH_2_O), acetone (C_3_H_6_O), and ethanol (C_2_H_6_O). The MoS_2_ layers exfoliated with both the grinding solvents showed good stability in three continuous response–recovery cycles at room temperature. Both of them completed a response–recovery cycle in 40 s and returned completely each time with almost no drift.

**Figure 5:**
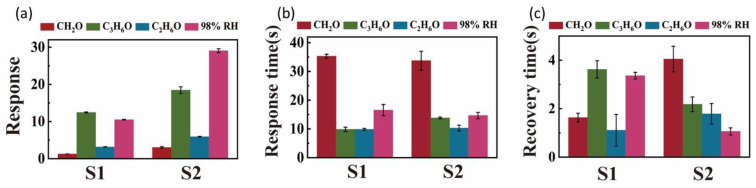
(**a**) Average responses; (**b**) response times; and (**c**) recovery times corresponding to the sensing curves of S1 and S2.

Figure 5 shows the average response, response time, and recovery time of S1 and S2 for the target analyte. As can be seen from Figure 5a, the sensitivities of MoS_2_ nanosheets exfoliation with NMP (S2) were 2.4, 1.4, 1.9, and 2.7 times higher than exfoliation with ACN (S1) to CH_2_O, C_3_H_6_O, C_2_H_6_O, and 98%RH, respectively. These results prove that the MoS_2_ nanosheets obtained by grinding with NMP have higher gas-responsive properties than the MoS_2_ nanosheets with ACN although NMP was not removed completely. At the same time, it can be seen from Figure 5b,c that both samples have faster response time to the four analytes, which did not exceed 35 s, and the recovery time did not exceed 4 s.

**Figure 6:**
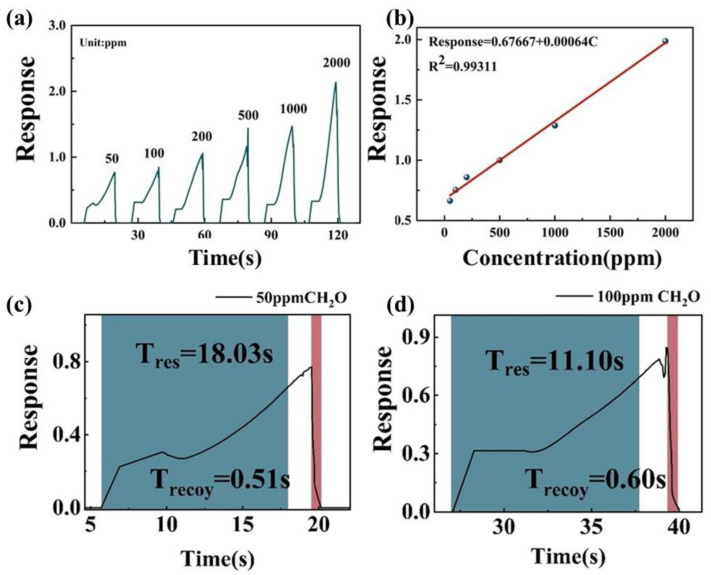
(**a**) Relationship between S2 response to CH_2_O at room temperature and the vapor concentration. (**b**) Fitted plot of response concentration of CH_2_O. (**c**,**d**) Response–recovery times of S2 at 50 ppm and 100 ppm, respectively.

In order to further evaluate the real-time monitoring capability of MoS_2_ nanosheets obtained by grinding with NMP (S1), the responses of the S2-based sensor under different concentrations (50–2000 ppm) of CH_2_O vapor were evaluated (Figure 6a). The response of S2 increased with the increase of CH_2_O concentration. Figure 5b shows a linear response to changing CH_2_O concentration, and the correlation coefficient R2 was 0.99, which facilitated gas-sensing application. Figure 6a,b show that the response time and recovery time of S2 were only 18 s and 0.5 s to 50 ppm CH_2_O, respectively, and only 11 s and 0.6 s to 100 ppm CH_2_O.

In order to comprehensively evaluate the gas-sensing performance of MoS_2_ nanosheets obtained by grinding with NMP, the performances of the MoS_2_ nanosheet-based sensors were compared (Table 1). As shown in Table 1, the response time and recovery time of MoS_2_ nanosheets obtained by grinding with NMP for 50 ppm CH_2_O were 18 s and 0.51 s, respectively, which were close to the shortest response time (11 s) and recovery time (8 s) shown by ZnS and In_2_O_3_/MoS_2_ [42,43]. Nevertheless, compared with the operating temperature (295 °C) of ZnS, the operating temperature of MoS_2_ nanosheets was at room temperature (25 °C). Therefore, the MoS_2_ nanosheets exhibited a robust sensing performance at a low working temperature, with rapid response and recovery. However, the sensitivity and limit of detection (LoD) of the sensor based on pure MoS_2_ nanosheets need to be improved.

**Figure 7:**
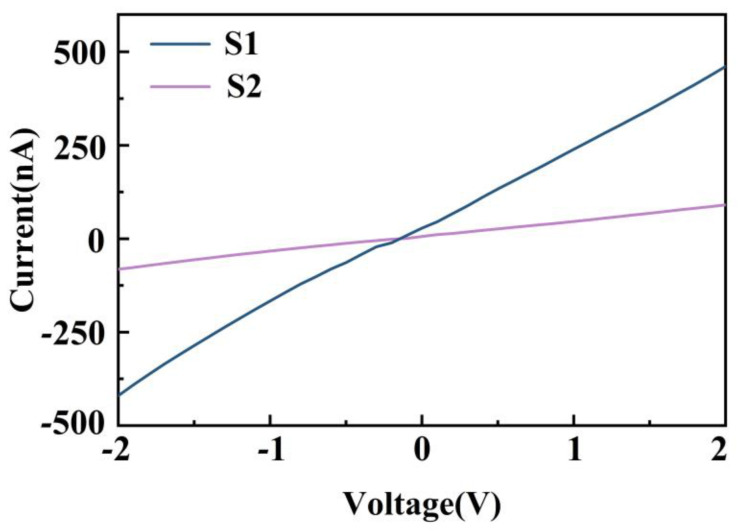
I–V curve of sensor based on MoS_2_ at 1000 ppm CH_2_O.

Figure 7 shows the I–V curves of S1 and S2 measured with an applied bias voltage ranging from −2 to 2 V at 1000 ppm CH_2_O. The I–V curves demonstrated a good ohmic contact between the sensing layers and the electrodes for both samples, which indicates that the sensor response was attributed to the sensitive material and not the metal–semiconductor contact.

The conductivity of the sensing material depends on the adsorption and desorption of gas molecules on the surface. When the MoS_2_ sensor is exposed to air, the oxygen molecules are adsorbed on the surface of the MoS_2_ nanosheets. Because of the strong electronegativity of oxygen atom, the adsorbed oxygen molecule captures electrons from the conduction bands of MoS_2_ nanosheets and generates ionized oxygen radicals, such as $O_{2}^{-},\;O^{-}$, and $O^{2-}$ [51]:

(1) $$O_{2}\mathrm{gas}\to O_{2}\mathrm{ads}$$

(2) $$O_{2}\mathrm{ads}+e^{-}\to O_{2}^{-}\left(100\;{}^{\circ}C\right)$$

(3) $$O_{2}^{-}\mathrm{ads}+e^{-}\to 2O^{-}\left(100-300\;{}^{\circ}C\right)$$

(4) $$O^{-}\mathrm{ads}+e^{-}\to O^{2-}(>300\;{}^{\circ}C)$$

**Figure 8:**
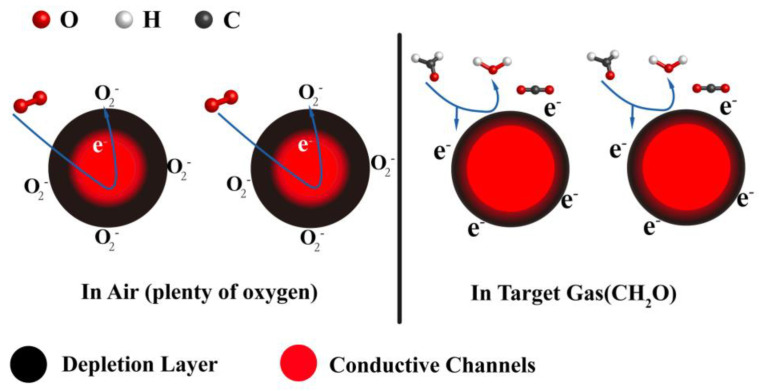
Schematic illustration of the sensing mechanism of MoS_2_ before and after exposure to target gas.

The sensing mechanism of MoS_2_ nanosheet to CH_2_O, C_3_H_6_O, C_2_H_6_O, and 98%RH have been well-studied and described elsewhere [52,53,54,55]. According to these references, MoS_2_-nanosheets-based gas sensors exhibit n-type characteristics in our work. The possible sensing mechanism is as follows: The transfer of electrons from the conduction band to chemisorbed oxygen decreases the carrier density and increases the depletion layer, thereby increasing the resistance of the MoS_2_ nanosheets. At room temperature, when the MoS_2_-nanosheet-based sensor is exposed to the target gas, for example, CH_2_O, the gas is adsorbed on the surface of the MoS_2_ nanosheets. These chemisorbed molecules react with $O_{2}^{-}$ (ads) to form H_2_O and CO_2_. Therefore, the trapped electrons are released back into the MoS_2_ nanosheets, which increases the number of conductive channels, leading to a decrease in sensor resistance (Figure 8).

## 4. Conclusions

MoS_2_ nanosheets were prepared with two grinding solvents via grinding-assisted liquid-phase exfoliation. The effects of grinding solvents on the structure of MoS_2_ nanosheets as well as the gas-sensing performance were studied. The structural and gas-sensing properties of MoS_2_ were investigated using XRD, SEM, and Raman spectroscopy. The sensing performance of MoS_2_ toward four target gases, including CH_2_O, C_3_H_6_O, C_2_H_6_O, and 98% RH, was analyzed at room temperature. The experimental results proved that the MoS_2_ nanosheets exfoliated with NMP responded better than the MoS_2_ nanosheets exfoliated with ACN although NMP was not removed completely. The MoS_2_ nanosheet-based sensor also exhibited excellent response. However, the sensitivity and LoD of the sensor need to be improved. Accordingly, although NMP cannot be removed completely from the surface of MoS_2_, NMP exhibits good gas sensitivity compared with other materials. Therefore, NMP is preferred for the preparation of gas-sensitive materials in grinding-assisted liquid-phase exfoliation. The results provide an experimental basis for the preparation of two-dimensional materials and their application in gas sensors.

## Figures and Tables

**Table 1:** Sensing performances of recently reported CH_2_O sensors.

Materials	Structure	Sensor Types	Con. (ppm)	Response (%)	LoD (ppb)	Temperature (°C)	Response Time (s)	Recovery Time (s)	Ref.
ZnS	0D nanosphere	Resistance	50	9440	-	295	11	8	[42]
In_2_O_3_/MoS_2_	Nanocubes/nonfilm	Resistance	50	75	200	RT	14	22	[43]
In_2_O_3_	Nanospheres	Resistance	1	3.5	1000	180	180	1000	[44]
In_2_O_3_/WS_2_	Nanocomposites	Resistance	5	7.5	-	RT	98	137	[45]
Au/TiO_2_	Hybrid films	Resistance	5	8.5	100	RT	36	110	[46]
rGo/MoS_2_	Hybrid films	Resistance	10	2.8	-	RT	-	-	[47]
Ni-doped In_2_O_3_/WS_2_	Nanocomposites	Resistance	20	32	150	RT	76	123	[45]
rGO/SnO_2_	Nanocomposites	Resistance	0.5	3200	10	125	31	62	[48]
MXene/NH_2_-MWCNTs	Hybrid films	Self-powered voltage	5	35	10	RT	51	57	[49]
MXene/ Co_3_O_4_	Hybrid films	Self-powered voltage	10	9.2	10	RT	83	5	[50]
MoS_2_	Nanosheets	Resistance	50	66.4	-	RT	18	0.5	This work

## Data Availability

The data presented in this study are available on request from the corresponding author.

## Supplementary Materials

The following are available online at https://www.mdpi.com/article/10.3390/nano12244485/s1, Figure S1: Schematic of sensing test system, Figure S2: The FWHM of XRD peak of the bulk MoS_2_, S1 and S2 from Figure 1. The FWHM of bulk MoS_2_, S1 and S2 are 0.1.31°, 0.1128°, 0.112°, respectively.

## References

[1] Hur J.H., Park J., Kim D., Jeon S. (2017). Model for the Operation of a Monolayer MoS_2_ Thin-Film Transistor with Charges Trapped near the Channel Interface. Phys. Rev. Appl..

[2] Fabbri F., Rotunno E., Cinquanta E., Campi D., Bonnini E., Kaplan D., Lazzarini L., Bernasconi M., Ferrari C., Longo M. (2016). Novel Near-infrared Emission from Crystal Defects in MoS_2_ Multilayer Flakes. Nat. Commun..

[3] Mak K.F., Lee C., Hone J., Heinz T.F. (2010). Atomically Thin MoS_2_: A New Direct-gap Semiconductor. Phys. Rev. Lett..

[4] Posudievsky O.Y., Khazieieva O.A., Cherepanov V.V., Dovbeshko G.I., Shkavro A.G., Koshechko V.G., Pokhodenko V.D. (2013). Improved Dispersant-free Liquid Exfoliation down to the Graphene-like State of Solvent-free Mechanochemically DelamiNated Bulk MoS_2_. J. Mater. Chem. C.

[5] Chang H.Y., Yogeesh M.N., Ghosh R., Rai A., Sanne A., Yang S., Lu N., Banerjee S.K., Akinwande D. (2016). Large-Area Monolayer MoS2 for Flexible Low-Power RF Nanoelectronics in the GHz Regime. Adv. Mater..

[6] Wang Q.H., Kalantar-Zadeh K., Kis A., Coleman J.N., Strano M.S. (2012). Electronics and Optoelectronics of Two-Dimensional Transition Metal Dichalcogenides. Nat. Nanotechnol..

[7] Radisavljevic B., Radenovic A., Brivio J., Giacometti V., Kis A. (2011). Single-Layer MoS2 Transistors. Nat. Nanotechnol..

[8] Lee G.H., Yu Y.J., Cui X., Petrone N., Lee C.H., Choi M.S., Lee D.Y., Lee C., Yoo W.J., Watanabe K. (2013). Flexible and Transparent MoS_2_ Field-Effect Transistors on Hexagonal Boron Nitride-Graphene Heterostructures. ACS Nano.

[9] Zhang D., Wu J., Li P., Cao Y. (2017). Room-temperature SO_2_ Gas Sensing Properties Based on Metal-doped MoS_2_ Nanoflower:An Experimental and Density Functional Theory Investigation. J. Mater. Chem. A.

[10] Zhang D., Sun Y., Li P., Zhang Y. (2016). Facile Fabrication of MoS_2_-modified SnO_2_ Hybrid Nanocomposite for Ultrasensitive Humidity Sensing. ACS App. Mater. Inter..

[11] Zhang D., Wu J., Cao Y. (2019). Ultrasensitive H_2_S Gas Sensing Properties of CuO Nanorods/MoS_2_ Nanosheets Nanoheterostructure with Synergistic Effect. Sens. Actuators B Chem..

[12] Han S.W., Kwon H., Kim S.K., Ryu S., Yun W.S., Kim D.H., Hwang J.H., Kang J.S., Baik J., Shin H.J. (2011). Band-Gap Transition Induced by Interlayer van Der Waals Interaction in MoS_2_. Phys. Rev. B.

[13] Cheng R., Jiang S., Chen Y., Liu Y., Weiss N., Cheng H.C., Wu H., Huang Y., Duan X. (2014). Few-Layer Molybdenum Disulfide Transistors and Circuits for High-Speed Flexible Electronics. Nat. Commun..

[14] Splendiani A., Sun L., Zhang Y., Li T., Kim J., Chim C.-Y., Galli G., Wang F. (2010). Emerging Photoluminescence in Monolayer MoS_2_. Nano Lett..

[15] Varghese S.S., Varghese S.H., Swaminathan S., Singh K.K., Mittal V. (2015). Two Dimensional Materials for Sensing: Graphene and Beyond. Electronics.

[16] Kumar M., Agrawal A.V., Moradi M., Yousef R., Abdeltif A., Assadi A.A., Nguyen-Tri P. (2020). Chapter 6—Nanosensors for Gas Sensing Applications. Nanomaterials for Air Remediation.

[17] Wypych F., Schöllhorn R. (1992). 1T-MoS_2_, A New Metallic Modifcation of Molybdenum Disulfde. J. Chem. Soc. Chem. Commun..

[18] Eda G., Yamaguchi H., Voiry D., Fujita T., Chen M., Chhowalla M. (2011). Photoluminescence from Chemically Exfoliated MoS_2_. Nano Lett..

[19] Agrawal A.V., Kumar N., Kumar M. (2021). Strategy and Future Prospects to Develop Room-Temperature-Recoverable NO_2_ Gas Sensor Based on Two-Dimensional Molybdenum Disulfide. Nano-Micro Lett..

[20] Zhao W., Ribeiro R.M., Eda G. (2015). Electronic Structure and Optical Signatures of Semiconducting Transition Metal Dichalcogenide Nanosheets. Acc. Chem. Res..

[21] Barzegar M., Iraji-zad A., Tiwari A. (2019). On the Performance of Vertical MoS_2_ Nanoflakes as a Gas Sensor. Vacuum.

[22] Ansari S.A., Fouad H., Ansari S.G., Pallashuddin Sk M., Cho M.H. (2017). Mechanically Exfoliated MoS_2_ Sheet Coupled with Conductive Polyaniline as a Superior Supercapacitor Electrode Material. J. Colloid Interface Sci..

[23] Joensen P., Frindt R.F., Morrison S.R. (1986). Single-layer MoS_2_. Mater. Res. Bull..

[24] Halim U., Chu R.Z., Yu C. (2013). A Rational Design of Cosolvent Exfoliation of Layered Materials by Directly Probing Liquid-solid Interaction. Nat. Commun..

[25] Yao Y., Tolentino L., Yang Z. (2013). High-Concentration Aqueous Dispersions of MoS_2_. Adv. Funct. Mater..

[26] Kim H.-S., Kumar M.D., Kim J.D. (2018). Lim Vertical Growth of MoS_2_ Layers by Sputtering Method for Efficient Photoelectric Application. Sens. Actuators A Phys..

[27] Goni F., Chemelli A., Uhlig F. (2021). High-Yield Production of Selected 2D Materials by Understanding Their Sonication-Assisted Liquid-Phase Exfoliation. Nanomaterials.

[28] Lee C.-S., Shim S.J., Kim T.H. (2020). Scalable Preparation of Low-Defect Graphene by Urea-Assisted Liquid-Phase Shear Exfoliation of Graphite and Its Application in Doxorubicin Analysis. Nanomaterials.

[29] Coleman J.N., Lotya M., O’Neill A., Bergin S.D., King P.J., Khan U., Young K., Gaucher A., De S., Smith R.J. (2011). Two-Dimensional Nanosheets Produced by Liquid Exfoliation of Layered Materials. Science.

[30] Zhou K.G., Mao N.N. (2011). A Mixed-Solvent Strategy for Efficient Exfoliation of Inorganic Graphene Analogues. Angew. Chem. Int. Ed..

[31] Hernandez Y., Nicolosi V., Lotya M., Blighe F.M., Sun Z., De S., McGovern I.T., Holland B., Byrne M., Gun’ko Y.K. (2008). High-Yield Production of Graphene by Liquid-Phase Exfoliation of Graphite. Nat. Nanotechnol..

[32] Tang Q., Zhou Z. (2013). Graphene-Analogous Low-Dimensional Materials. Prog. Mater. Sci..

[33] Li Y., Yin X., Wu W. (2018). Preparation of Few-Layer MoS_2_ Nanosheets via an Efficient Shearing Exfoliation Method. Ind. Eng. Chem. Res..

[34] Emily P.N., Benjamin J.C., Torben D., Jian Z.O., Kay L., Serge Z., Kourosh K. (2015). Investigation of Two-solvent Grinding-assisted Liquid Phase Exfoliation of Layered MoS_2_. Chem. Mater..

[35] Xia Y., Wu Z., Qin Z., Chen F., Lv C., Zhang M., Shaymurat T., Duan H. (2022). Wool-Based Carbon Fiber/MoS_2_ Composite Prepared by Low-Temperature Catalytic Hydrothermal Method and Its Application in the Field of Gas Sensors. Nanomaterials.

[36] Lin Y., Liu K., Chen Y., Liu L. (2014). Influence of Graphene Functionalized with Zine Dimethacrylate on the Mechanical and Thermal Properties of Natural Rubber Nanocomposites. Polym. Compos..

[37] Shao L., Wu Z., Duan H., Talgar S. (2018). Discriminative and rapid detection of ozone realized by sensor array of Zn^2+^ doping tailored MoS_2_ ultrathin nanosheets. Sens. Actuators B-Chem..

[38] Cho S.Y., Kim S.J., Lee Y., Kim J.S., Jung W.B., Yoo H.W., Kim J., Jung H.-T. (2015). Highly Enhanced Gas Adsorption Properties in Vertically Aligned MoS_2_ Layers. ACS Nano.

[39] Xie J., Zhang J., Li S., Grote F., Zhang X., Zhang H., Wang R., Lei Y., Pan B., Xie Y. (2013). Controllable Disorder Engineering in Oxygen-Incorporated MoS_2_ Ultrathin Nanosheets for Efficient Hydrogen Evolution. J. Am. Chem. Soc..

[40] Lin H., Wang J., Luo Q., Peng H., Luo C., Qi R., Huang R., Travas-Sejdic J., Duan C. (2017). Rapid and Highly Efficient Chemical Exfoliation of Layered MoS_2_ and WS_2_. J. Alloys Compd..

[41] Lee C., Yan H., Lee C., Yan H., Brus L.E., Heinz T.F., Hone J., Ryu S. (2010). Anomalous Lattice Vibrations of Single-and Few-layer MoS_2_. ACS Nano.

[42] Hussain S., Liu T., Javed M.S., Aslam N., Zeng W. (2017). Highly Reactive 0D ZnS Nanospheres and Nanoparticles for Formaldehyde Gas-sensing Properties. Sens. Actuators B Chem..

[43] Cao J., Zhang N., Yang S., Xu W., Zhang X., Zhang H., Wang S. (2022). Study on the Selectivity Difference of Formaldehyde and Ethanol Induced by Variation of Energy Gap in In_2_O_3_ Hierarchical Materials. Colloids Surf. A..

[44] Zhang D., Jiang C., Wu J. (2018). Layer-by-layer Assembled In_2_O_3_ Nanocubes/flower-like MoS_2_ Nanofilm for Room Temperature Formaldehyde Sensing. Sens. Actuators B Chem..

[45] Zhang D., Cao Y., Yang Z., Wu J. (2020). Nanoheterostructure Construction and DFT Study of Ni-doped In_2_O_3_ Nanocubes/WS_2_ Hexagon Nanosheets for Formaldehyde Sensing at Room Temperature. ACS Appl. Mater. Interfaces.

[46] Zhang S., Zhao L., Huang B., Li X. (2022). Enhanced Sensing Performance of Au-decorated TiO_2_ Nanospheres with Hollow Structure for Formaldehyde Detectionn at Room Temperature. Sens. Actuators B.

[47] Li X., Wang J., Xie D., Xu J., Xia Y., Li W., Xiang L., Li Z., Xu S., Komarneni S. (2017). Flexible Room-temperature Formaldehyde Sensors Based on rGO Film and rGo/MoS_2_ Hybrid film. Nanotechnology.

[48] Shanmugasundaram A., Manorama S.V., Kim D.S., Jeong Y.J., Lee D.W. (2022). Toward Point-of-care Chronic Disease Management: Biomarker Detection in Exhaled Breath Using an E-Nose Sensor Based on rGO/SnO_2_ Superstructures. Chem. Eng. J..

[49] Wang D., Zhang D., Chen X., Zhang H., Tang M., Wang J. (2022). Multifunctional Respiration-driven Triboelectric Nanogenerator for Self-powered Detection of Formaldehyde in Exhaled Gas and Respiratory Behavior. Nano Energy.

[50] Zhang D., Mi Q., Wang D., Li T. (2021). MXene/Co_3_O_4_ Composite Based Formaldehyde Sensor Driven by ZnO/MXene Nanowire Arrays Piezoelectric Nanogenerator. Sens. Actuators B.

[51] Xiao C., Liu C., Li L., Yu L., Wang Z., Bo X. (2014). Tungsten Trioxide Nanotubes with High Sensitive and Selective Properties to Acetone. Sens. Actuators B Chem..

[52] Cho B., Hahm M.G., Choi M., Yoon J., Kim A., Lee Y.J., Park S., Kwon J., Kim C., Song M. (2015). Charge-transfer-based Gas Sensing Using Atomic-layer MoS_2_. Sci. Rep..

[53] Liu X., Ma T., Pinna N., Zhang J. (2017). Two-Dimensional Nanostructured Materials for Gas Sensing. Adv. Funct. Mater..

[54] Zhou Q., Wu H., Wang C., Zhang S., Zhang L., Zhang H., Chen J., Pan G. (2020). Ultrasonic-triggered Surface Morphological Reconstruction of MoS_2_ for Enhanced Ultrasensitive Humidity Sensing. ChemNanoMat.

[55] Tan Y., Yu K., Yang T., Zhang Q., Cong W., Yin H., Zhang Z., Chen Y., Zhu Z. (2014). The Combinations of Hollow MoS2 Micro@nano-spheres: One-step Synthesis, Excellent Photocatalytic and Humidity Sensing Properties. J. Mater. Chem. C.

