# Supplementary material for: Effect of Different Solvents on Morphology and Gas-Sensitive Properties of Grinding-Assisted Liquid-Phase-Exfoliated MoS2 Nanosheets
Source: Nanomaterials (Basel). 2022 Dec 18;12(24):4485. doi: 10.3390/nano12244485 (PMC9784282; doi:10.3390/nano12244485)
Supplement: Supplementary file 1 [file nanomaterials-12-04485-s001.zip › nanomaterials-2056784-supplementary.pdf]

Supplementary Information

Effect of Different Solvents on Morphology and Gas-Sensitive Properties of Grinding-Assisted Liquid Phase-Exfoliated MoS_2 Nanosheets

Hao Wang [1,2], Xiaojie Xu [1,2] and Talgar Shaymurat [1,2,*]

[1] Key Laboratory of New Energy and Materials Research, Xinjiang Institute of Engineering, Urumqi 830023, China
[2] Xinjiang Condensed Matter Phase Transition and Microstructure Laboratory, College of Physics Science and Technology, Yili Normal University, Yining 835000, China
*Correspondence: talgar.shaymurat@vip.163.com

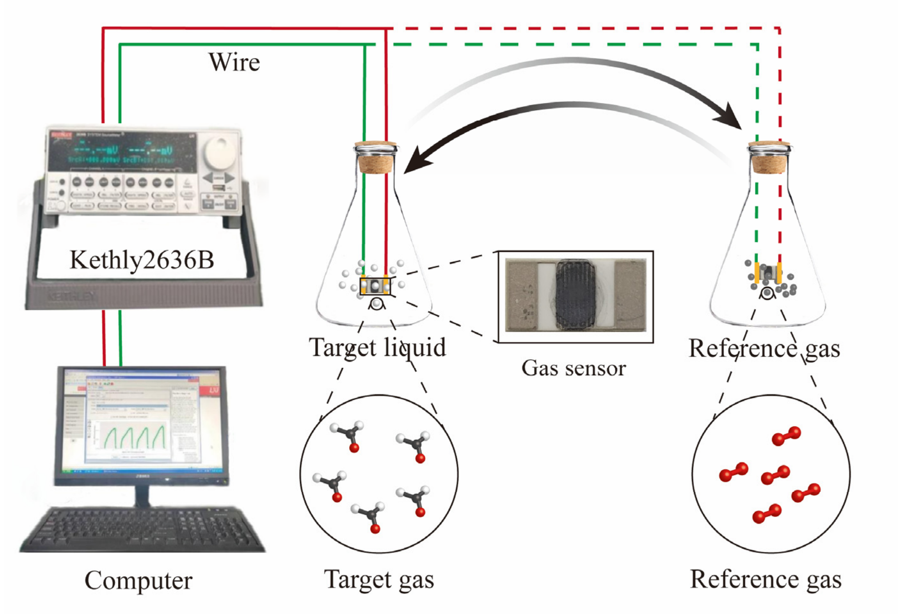

Figure S1. Schematic of sensing test system.

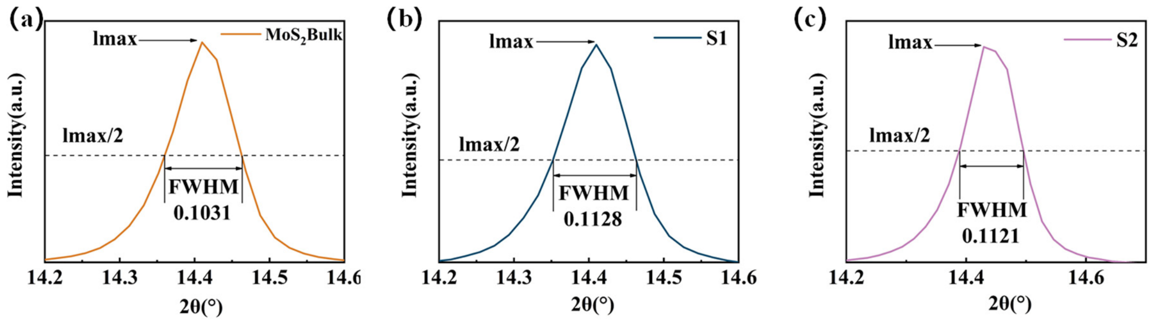

Figure S2. The FWHM of XRD peak of the bulk MoS_2, S1 and S2 from Figure 1. The FWHM of bulk MoS_2, S1 and S2 are 0.1.31°, 0.1128°, 0.112°, respectively.
